# Did COVID-19 lockdown delay actually worsen melanoma prognosis?^☆^

**DOI:** 10.1016/j.abd.2022.08.004

**Published:** 2022-12-08

**Authors:** Pedro Gil-Pallares, Olalla Figueroa-Silva, Maria Eugenia Gil-Pallares, José Ángel Vázquez-Bueno, Francisca Piñeyro-Molina, Benigno Monteagudo, Cristina De las Heras-Sotos

**Affiliations:** a Department of Dermatology, Complejo Hospitalario Universitario de Ferrol, Ferrol, Spain; b Department of Pathology, Complejo Hospitalario Universitario de Ferrol, Ferrol, Spain; c Universidad de Santiago de Compostela, Santiago de Compostela, Spain

**Keywords:** Melanoma, COVID-19, Prognosis, Quarantine, SARS-CoV-2

## Abstract

**Background:**

The COVID-19 lockdown possibly meant a delay in the diagnosis and treatment of melanoma and therefore, worsening its prognosis. This unique situation of diagnosis deferral is an exceptional opportunity to investigate melanoma biology.

**Objectives:**

To evaluate the immediate and mid-term impact of diagnosis delay on melanoma.

**Methods:**

A retrospective observational study of melanoma diagnosed between March 14^th^ 2019 and March 13^th^ 2021. We compared the characteristics of melanomas diagnosed during the first 6-month period after the lockdown instauration and a second period after recovery of normal activity with the same periods of the previous year, respectively.

**Results:**

A total of 119 melanomas were diagnosed. There were no differences in age, sex, incidence, location, presence of ulceration or mitoses, and in situ/invasive melanoma rate (p > 0.05). After the recovery of the normal activity, Breslow thickness increased in comparison with the previous year (2.4 vs 1.9 mm, p < 0.05) resulting in a significant upstaging according to the AJCC 8^th^ ed. (p < 0.05).

**Study limitations:**

The main limitation is that this is a single-center study.

**Conclusions:**

The COVID-19 lockdown implied a diagnosis delay leading to a mid-term increase in Breslow thickness and an upstaging of invasive melanomas. However, the detection deferral did not result in a higher progression of in situ to invasive melanoma, in our sample.

## Introduction

Due to the Coronavirus Disease 2019 (COVID-19) pandemic, many countries went into lockdown, which began in Spain on March 14^th^ 2020.^1^ Most surgeries and appointments were canceled, and the population was concerned about getting infected. Therefore, patients are mainly consulted for severe symptoms. This could lead to a reduction in the diagnosis of different diseases such as skin cancer,^2^, ^3^ heart attacks,^4^ or strokes,^5^ and a compromise of cancer screening programs.^6^, ^7^ In the case of melanoma, a recent study estimated an upstaging of 45% of invasive melanomas after a 3-month delay.^8^

Although some authors noted a reduction in the number of diagnosed melanomas during the lockdown,^2^, ^9^ other studies showed the opposite results.^10^, ^11^ However, since most articles limited the studied period to the lockdown it is only possible to estimate the impact of the delay in melanoma diagnosis. We compared the melanomas diagnosed after the lockdown, not only short-term but also once the normal clinical activity was restored, in order to assess if the diagnosis delay due to the COVID-19 lockdown actually meant a prognosis worsening of the melanomas.

## Methods

A retrospective, single-center, observational study of histopathologically diagnosed melanomas between March 14^th^ 2019, and March 13^th^ 2021 was designed. This study was approved by the Institutional Ethics Committee. The data was retrieved from the Pathology Department records of our hospital. Demographic data, referral via teledermatology, melanoma location, Breslow thickness (mm), presence of mitosis and ulceration, and AJCC (American Joint Committee on Cancer 8^th^ edition) staging were collected. Incidence was calculated per 100.000 inhabitants. Metastatic melanoma and clinically diagnosed melanoma without histopathological confirmation were excluded. The first 6-month period following the start of the lockdown, between March 14^th^ 2020, and September 13^th^ 2020 (1^st^ P20), and a second 6-month period between September 14^th^ 2020, and March 13^th^ 2021 (2^nd^ P20) were compared with the same periods of the previous year (1^st^ P19 and 2^nd^ P19, respectively).

Statistical analysis was performed using R (R Core Team, Vienna, Austria); *t*-test and *U*-Mann Whitney were used for quantitative variables. Fisher’s exact test was used for the analysis of qualitative variables.

## Results

A total of 119 melanomas were included. The characteristics of melanomas are summarized in Table 1. A total of 29 and 24 melanomas were diagnosed in the 1^st^ P19 and 1^st^ P20 respectively, and no differences were found in the incidence (16.1 vs. 13.3 per 100.000 inhabitants, p > 0.05). A total of 36 and 30 melanomas were diagnosed in the 2^nd^ P19 and 2^nd^ P20 respectively, also without differences in the incidence (20 vs. 16.7 per 100.000 inhabitants, p > 0.05).

**Table 1:** Patient and tumor characteristics of the melanomas diagnosed between March 14^th^ 2019 and March 13^th^ 2021.

	1^st^ Period			2^nd^ Period		
	1^st^ P19	1^st^ P20	p-value	2^nd^ P19	2^nd^ P20	p-value
	n = 29	n = 24		n = 36	n = 30	
**Incidence per-100.000 inhabitants**			0.583			0.539
	16.1	13.3		20	16.7	
**Age (mean)**			0.162			0.974
	59 ± 18	66 ± 17		70 ± 17	69 ± 16	
**Sex**			1			0.612
Male	14 (48%)	11 (46%)		11 (31%)	11 (37%)	
Female	15 (52%)	13 (54%)		25 (69%)	19 (63%)	
**Location**			0.777			0.396
Head and neck	10 (34%)	7 (29%)		12 (33%)	9 (30%)	
Anterior trunk	2 (7%)	4 (17%)		2 (6%)	1 (3%)	
Posterior trunk	9 (31%)	7 (29%)		11 (31%)	8 (27%)	
Upper extr.	6 (21%)	3 (13%)		4 (11%)	2 (7%)	
Lower extr.	2 (7%)	2 (8%)		7 (19%)	6 (20%)	
Acral	0	1 (4%)		0	4 (13%)	
**Referral**			0.250			1
Teledermatology	8 (28%)	11 (46%)		10 (28%)	8 (27%)	
***In situ*/invasive**			0.166			0.458
*In situ*	16 (55%)	8 (33%)		15 (42%)	16 (53%)	
Invasive	13 (45%)	16 (67%)		21 (58%)	14 (47%)	
**Breslow Thickness (mm, mean)**			0.263			0.040^a^
	1.8 ± 1.7	1.2 ± 1.4		1.9 ± 2.7	2.4 ± 2.3	
**Ulceration**			1			0.453
	3 (10%)	3 (12%)		3 (8%)	5 (17%)	
**Mitoses**			0.767			0.310
	8 (28%)	8 (33%)		10 (28%)	12 (40%)	
**pT staging group (AJCC 8^th^ ed.)**			0.770			0.044^a^
pT1a	5 (17%)	9 (38%)		14 (39%)	6 (20%)	
pT1b	1 (3%)	2 (8%)		1 (3%)	1 (3%)	
pT2a	4 (14%)	1 (4%)		0	0	
pT2b	1 (3%)	1 (4%)		0	0	
pT3a	1 (3%)	1 (4%)		0	3 (10%)	
pT3b	0	1 (4%)		0	2 (7%)	
pT4a	0	0		3 (8%)	0	
pT4b	1 (3%)	1 (4%)		3 (8%)	2 (7%)	

Footnote: 1^st^ P19, March 14^th^ 2019 ‒ September 13^th^ 2019. 1^st^ P20, March 14^th^ 2020 – September 13^th^ 2020. 2^nd^ P19, September 14^th^ 2019 ‒ March 13^th^ 2020. 2^nd^ P20, September 14^th^ 2020 ‒ March 13^th^ 2021.

a Statistical significance.

The patients in both periods were similar in age and sex (p > 0.05). There were no differences in location, teledermatology referral rate, and presence of ulceration or mitosis (p > 0.05).

Invasive melanomas represented 45% and 67% of the diagnosed melanomas in 1^st^ P19 and 1^st^ P20 respectively. After the recovery of the normal activity, the proportion of invasive melanomas was 58% for 2^nd^ P19 and 47% for 2^nd^ P20. However, no statistically significant differences in in situ/invasive melanoma rates were found in any of the periods.

**Figure 1:**
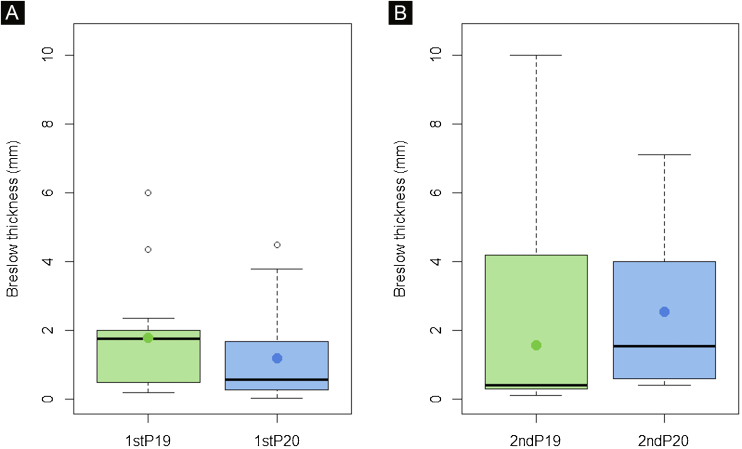
Breslow thickness of invasive melanomas did not significantly differ during the first period (p > 0.05) (A). However, a statistically significant increase in Breslow thickness was seen in the 2^nd^ P20, after the recovery of normal activity, in comparison with the previous year (p < 0.05) (B). These results suggest that some invasive melanomas suffered a diagnosis delay which implied a prognosis worsening.

Breslow thickness of diagnosed melanomas increased in 2ndP20 in comparison with the previous year (2.4 vs. 1.9 mm, p < 0.05) which implied differences in the AJCC stage (p < 0.05). However, no differences were found in the first period (p > 0.05) (Fig. 1).

## Discussion

Different approaches, such as promoting teledermatology,^12^ were considered to minimize the effects of COVID-19 on melanoma diagnosis. Valenti et al.^13^ reported a 2.3-month postponement of revisions of advanced skin cancer, which is similar to the 3-mont average delay that we authors estimated since it took 6-months from the beginning of the lockdown until the normal clinical activity was achieved again. According to some studies, this delay would be enough to observe an upstaging of the invasive melanomas,^8^ but this increase in Breslow thickness would only be seen once the delay occurred instead of during the lockdown. For this reason, we included a second period after the clinical activity recovered pre-lockdown levels in order to analyze if the diagnosis delay actually had a repercussion in those melanomas that potentially “stayed at home”.

Some authors found a significant reduction in melanoma diagnosis during the lockdown.^2^, ^9^ However, similar to other reports,^10^, ^11^ although we found a decrease in the number of melanomas in both periods (5 in the first period and 6 in the second period), we did not find a significant reduction in the incidence of melanomas per 100.000 inhabitants in any period.

Some reports showed an increase in Breslow thickness or an upstaging of the diagnosed melanomas during the lockdown in comparison with the previous year,^14^, ^15^ but since some also showed an important decrease in the number of diagnoses, mainly of in situ melanomas,^15^ we believe that it would be the outcome of thin melanoma under-diagnosis rather than an “immediate” growth of melanomas due to the lockdown. As expected, we did not find differences in Breslow thickness in the first period, but we found a significant increase in Breslow thickness after the recovery of the normal activity compared with the previous year (1.9 mm in 2^nd^ P19 vs. 2.4 mm in 2^nd^ P20, p < 0.05) (Fig. 1), which implied an upstaging according to the AJCC 8^th^ ed. (p < 0.05). This indicates that although we did not find a reduction in the incidence, some melanomas actually suffered a diagnosis delay.

However, this prognosis worsening found in invasive melanomas due to the deferral seems not to affect in situ melanomas likewise. Coinciding with other reports,^10^ our results do not show differences in in situ and invasive melanoma rates before and after the beginning of the lockdown, and after the recovery of normal clinical activity (p > 0.05). Therefore, we can assume that a 3-month average diagnosis delay due to COVID-19 did not mean a progression of in situ melanomas in our sample.

More studies after the normal clinical activity was resumed are required to assess if the diagnosis deferral actually implied an increased progression to invasive melanomas. Although this situation of melanoma diagnosis delay will hopefully not happen again, we believe that it is an exceptional opportunity for the investigation of in situ and invasive melanoma biology. Further investigation in this area could provide valuable information to the melanoma detection protocols and to better understand its natural behavior.

## Conclusions

It was not found a reduction in the incidence of melanomas in any of the considered periods. However, the increase in Breslow thickness and the consequent upstaging observed, once the normal clinical activity was restored, suggests that the COVID-19 lockdown caused a delay in the diagnosis of melanoma and a prognosis worsening of the invasive melanomas. The similar in situ/invasive melanoma rates suggest that a 3-month average diagnosis delay does not necessarily imply an increase in the progression of in situ melanomas.

We believe that it is important to reassess the real repercussion of the lockdown in melanoma by analyzing the melanomas diagnosed once the pre-pandemic clinical activity was restored. The presented results might not be completely generalizable to other regions with different COVID-19 incidences. Therefore, we encourage other authors to analyze mid-term melanoma diagnoses.

## Financial support

None declared.

## Authors' contributions

Pedro Gil-Pallares: The study concept and design; data collection, or analysis and interpretation of data; statistical analysis; writing of the manuscript or critical review of important intellectual content; data collection, analysis and interpretation; effective participation in the research guidance; intellectual participation in the propaedeutic and/or therapeutic conduct of the studied cases; critical review of the literature; final approval of the final version of the manuscript.

Olalla Figueroa-Silva: The study concept and design; data collection, or analysis and interpretation of data; statistical analysis; writing of the manuscript or critical review of important intellectual content; data collection, analysis and interpretation; effective participation in the research guidance; intellectual participation in the propaedeutic and/or therapeutic conduct of the studied cases; critical review of the literature; final approval of the final version of the manuscript.

Maria Eugenia Gil-Pallares: The study concept and design; data collection, or analysis and interpretation of data; statistical analysis; writing of the manuscript or critical review of important intellectual content; data collection, analysis and interpretation; effective participation in the research guidance; intellectual participation in the propaedeutic and/or therapeutic conduct of the studied cases; critical review of the literature; final approval of the final version of the manuscript.

José Ángel Vázquez-Bueno: The study concept and design; data collection, or analysis and interpretation of data; statistical analysis; writing of the manuscript or critical review of important intellectual content; data collection, analysis and interpretation; effective participation in the research guidance; intellectual participation in the propaedeutic and/or therapeutic conduct of the studied cases; critical review of the literature; final approval of the final version of the manuscript.

Francisca Piñeyro-Molina: The study concept and design; data collection, or analysis and interpretation of data; statistical analysis; writing of the manuscript or critical review of important intellectual content; data collection, analysis and interpretation; effective participation in the research guidance; intellectual participation in the propaedeutic and/or therapeutic conduct of the studied cases; critical review of the literature; final approval of the final version of the manuscript.

Benigno Monteagudo: The study concept and design; data collection, or analysis and interpretation of data; statistical analysis; writing of the manuscript or critical review of important intellectual content; data collection, analysis and interpretation; effective participation in the research guidance; intellectual participation in the propaedeutic and/or therapeutic conduct of the studied cases; critical review of the literature; final approval of the final version of the manuscript.

Cristina De las Heras-Sotos: The study concept and design; data collection, or analysis and interpretation of data; statistical analysis; writing of the manuscript or critical review of important intellectual content; data collection, analysis and interpretation; effective participation in the research guidance; intellectual participation in the propaedeutic and/or therapeutic conduct of the studied cases; critical review of the literature; final approval of the final version of the manuscript.

## Conflicts of interest

None declared.
